# Rapid response systems: a systematic review and meta-analysis

**DOI:** 10.1186/s13054-015-0973-y

**Published:** 2015-06-12

**Authors:** Ritesh Maharaj, Ivan Raffaele, Julia Wendon

**Affiliations:** Kings College London, Denmark Hill, London, SE5 9RW UK; Department of Critical Care Medicine, King’s College Hospital NHS Foundation Trust, Denmark Hill, London, SE5 9RW UK; Department of Critical Care Medicine, Kings College London, Ground Floor, Cheyne Wing, Denmark Hill, London, SE5 9RS UK

## Abstract

**Introduction:**

Although rapid response system teams have been widely adopted by many health systems, their effectiveness in reducing hospital mortality is uncertain. We conducted a meta-analysis to examine the impact of rapid response teams on hospital mortality and cardiopulmonary arrest.

**Method:**

We conducted a systematic review of studies published from January 1, 1990, through 31 December 2013, using PubMed, EMBASE, CINAHL (Cumulative Index to Nursing and Allied Health Literature) and the Cochrane Library. We included studies that reported data on the primary outcomes of ICU and in-hospital mortality or cardiopulmonary arrests.

**Results:**

Twenty-nine eligible studies were identified. The studies were analysed in groups based on adult and paediatric trials that were further sub-grouped on methodological design. There were 5 studies that were considered either cluster randomized control trial, controlled before after or interrupted time series. The remaining studies were before and after studies without a contemporaneous control. The implementation of RRS has been associated with an overall reduction in hospital mortality in both the adult (RR 0.87, 95 % CI 0.81–0.95, p<0.001) and paediatric (RR=0.82 95 % CI 0.76–0.89) in-patient population. There was substantial heterogeneity in both populations. The rapid response system team was also associated with a reduction in cardiopulmonary arrests in adults (RR 0.65, 95 % CI 0.61–0.70, p<0.001) and paediatric (RR=0.64 95 % CI 0.55–0.74) patients.

**Conclusion:**

Rapid response systems were associated with a reduction in hospital mortality and cardiopulmonary arrest. Meta-regression did not identify the presence of a physician in the rapid response system to be significantly associated with a mortality reduction.

**Electronic supplementary material:**

The online version of this article (doi:10.1186/s13054-015-0973-y) contains supplementary material, which is available to authorized users.

## Introduction

Many ward patients may deteriorate to the point of unexpected ICU admission or even cardiac arrest and death. About one-half of the serious adverse events are deemed to be preventable [1]. Patients often show some signs of physiological deterioration for several hours (median 6 hours) before cardiac arrest [2, 3]. Theoretically, this would appear to be sufficient time to deliver interventions that would alter the trajectory of deterioration. This ‘failure to rescue’ is the context in which rapid response systems (RRSs) have been introduced [4].

Most RRS trials have used similar criteria for activation of the team. These include various thresholds for respiratory rate, heart rate, blood pressure and mental state as well as the ‘staff worried’ criterion [5]. Unfortunately, the determination of respiratory rate and mental state on the wards is error prone and vital signs are unlikely to be measured more frequently than five times per day outside the ICU [6–8]. Variations in activation potentially influence the effectiveness of RRSs. Non-activation and delays in activation may be associated with harm and too liberal activation may cause system fatigue [9–11]. Consequently, the time spent implementing and maintaining the concept of a RRS and the rate of RRS activation may be a contributory factor to its success.

The RRS team is usually multidisciplinary in nature and tailored to meet the institutional needs and resources. In this respect there is some variation in the constitution of a RRS. In the UK the RRS team may be nurse led, and in the USA nurse or respiratory therapists may lead [12, 13]. In Australia, New Zealand and Scandinavia, a physician-led RRS is favoured [5, 14]. The optimal composition of a RRS team is unknown and may depend upon organizational structure.

Organizations such as the Institute for Healthcare Improvement, the 2009 Joint Commission’s National Patient Safety Goal in the USA, the National Institute of Clinical Excellence (NICE) in the UK as well as numerous other organizations have driven the implementation of RRS teams [15, 16]. Despite high face validity, the effectiveness of RRS teams in reducing hospital mortality remains controversial. A high-quality meta-analysis in 2010 did not find evidence of RRS teams reducing hospital mortality in adults [17]. A more recent review by Winters et al. [18] included studies of varying methodological quality and design, with inconsistent findings across outcomes. Both analyses aggregated studies of varying methodology and quality, further limiting the interpretation of the results [18].

Accordingly, a systematic review and meta-regression was undertaken to assess the effect of the RRS on hospital mortality and cardiopulmonary arrest outside the ICU and to evaluate the potential relationship between the number of RRS team activations per 1000 admissions, the presence of a physician in the RRS team and the duration of the implementation phase and the effectiveness of RRS teams.

## Methods

**Fig. 1:**
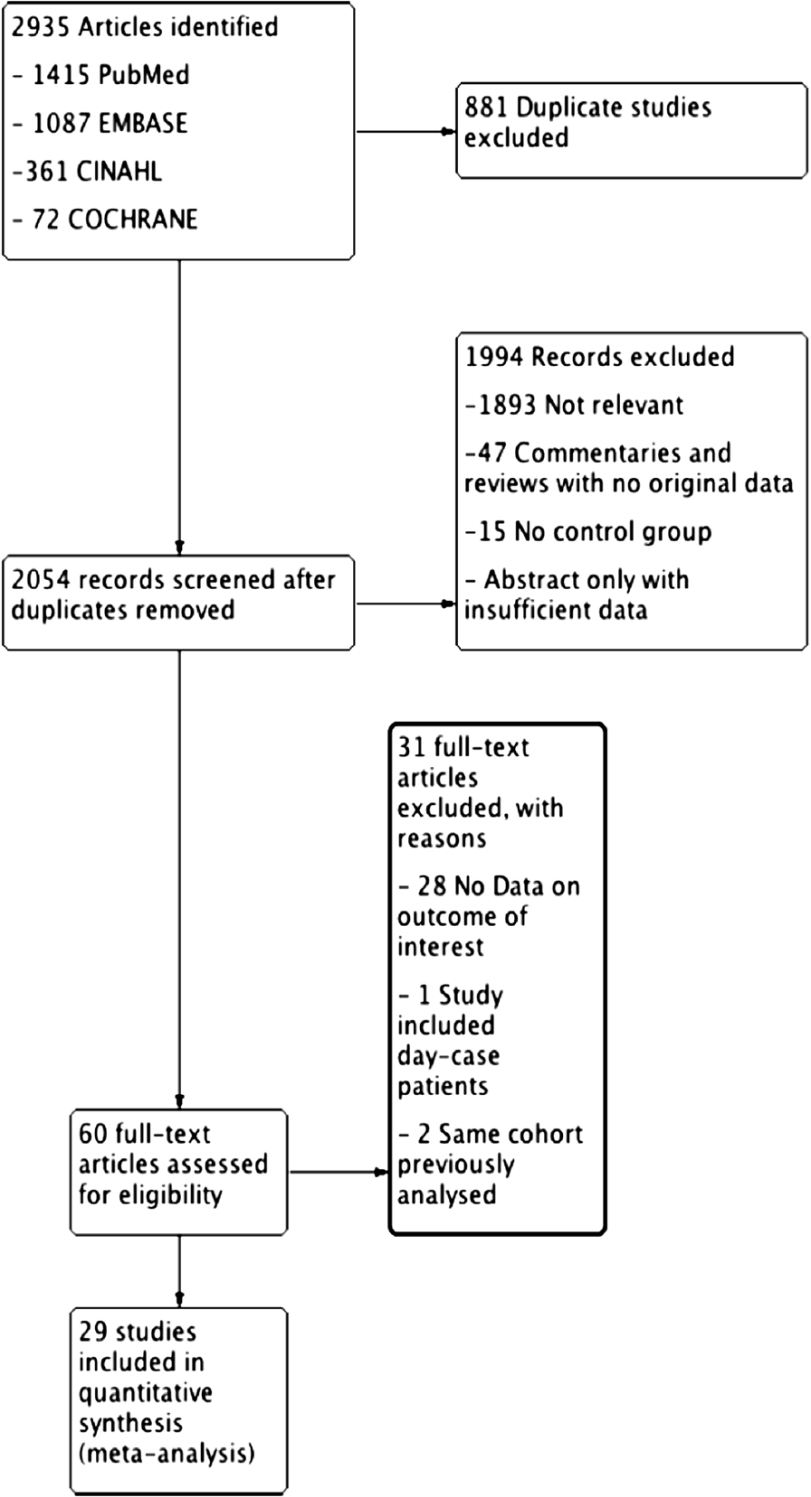
Literature search flow diagram

A systematic review of studies published between 1 January 1990 and 31 December 2013 was conducted in accordance with published guidelines [19, 20]. We used the PubMed, EMBASE, CINAHL (Cumulative Index to Nursing and Allied Health Literature) and Cochrane Register of Controlled trials databases. Additionally, a hand search of bibliographies of key publications was performed. Search terms included ‘rapid response team’, medical emergency team’ and ‘critical care outreach’. Details of the electronic search are described in Fig. 1 with additional information provided in the supplementary appendix.

### Study eligibility criteria and selection

The inclusion criteria for this meta-analysis was that studies had to be a study that described the effect of RRS teams in a population of hospital in-patients that included a comparison between a control cohort and intervention cohort, and provided quantitative data about mortality rates or cardiopulmonary arrests. There was no country restriction but only English language studies were included.

A total of 2935 abstracts were identified by the search strategy. The titles and abstracts were independently assessed for eligibility by two investigators (RM and IR). Eight hundred and eighty-one duplicate studies were removed and a further 1994 studies were removed because they were not relevant or did not report sufficient data (Fig. 1). In cases with multiple articles with overlapping data from the same population, we included data from the most comprehensive study and excluded the other studies [21–24].

### Data extraction process

Two reviewers independently, using a standardized format, extracted the data. For each study the following data elements were extracted: year of study, type of study design (randomized controlled trial (RCT), before–after, interrupted time series), type of hospital, number of centres, type of RRS, types of interventions, period pre and post implementation, demographics, number of activations per 1000 admissions, effect on do-not-resuscitate orders, non-ICU cardiopulmonary arrest, hospital mortality, ICU admissions as well as study quality indicators. Authors were contacted for additional data as required.

### Risk of bias in individual studies

Reviewers worked independently to assess study quality. The Newcastle Ottawa Scale (NOS) was used for assessing non-randomized studies [25]. The NOS uses a star system to evaluate the selection of study groups, the comparability of groups and the ascertainment of either the exposure or outcome of interest. The interrupted time series, controlled before–after and cluster randomized studies were evaluated using the criteria recommended by the Cochrane Effective Practice and Organisation of Care Group [26]. This included recruitment bias, baseline imbalance, loss of clusters, incorrect analysis or selective outcome reporting. To explore variability in study results (heterogeneity) we specified the following hypotheses *a priori*. We hypothesized that effect size may differ according to the number of activations per 1000 admissions, the duration of implementation in months and the presence of a physician on the team. The proposed standardized method to report the dose of RRS teams is activations per 1000 patient admissions [27].

### Data analysis

The primary outcome was hospital mortality. The secondary outcomes were non-ICU cardiopulmonary arrest and ICU admission rates. The meta-analyses were performed by computing the risk ratio (RR) using a random effects model. The random effects model provides more conservative estimates of treatment effects in the face of heterogeneity. The analysis was performed in subgroups of adults and paediatrics with further subgroups by study design (cluster randomized control trial (CRCT), interrupted time series and controlled before–after versus before–after studies with no contemporaneous controls and observational studies). Quantitative analysis was performed using an intention-to-treat analysis, and the RR and 95 % confidence intervals (CIs) were calculated [28]. We used the method proposed by Higgins et al. [29] to measure inconsistency between study results, reported as the *I*^2^ statistic as well as the 95 % CI. Publication bias was evaluated using the contour funnel plot asymmetry and the Harbord modification of the Egger test [30, 31]. We performed both analyses because graphical evaluation can be subjective. Factors other than publication bias can cause asymmetry in the funnel plot. These include factors such as study quality or true study heterogeneity.

A meta-regression was undertaken to explore the association between treatment effect and study characteristics [32]. These predefined factors included the number of activations per 1000 admissions, the presence of a physician in the RRS team and the time period for implementation.

The study by Hillman et al. [5] was a cluster randomized trial. The approach recommended by the Cochrane Collaboration to approximate the effective sample size is to divide by the design effect [20]. The design effect is calculated as:

$$ \mathrm{Design}\ \mathrm{effect} = 1 + \left(M\hbox{--}\ 1\right) \times \mathrm{I}\mathrm{C}\mathrm{C} $$

where *M* is the average cluster size and ICC is the intracluster correlation coefficient. The ICC compares the variance within clusters with the variance between clusters. Mathematically this is the between-cluster variability divided by the sum of the within-cluster and between-cluster variabilities. An ICC value of 0 gives a design effect of 1. This value indicates that individuals within a cluster have no similarity and there is no adjustment for sample size. An ICC of 1 indicates that all individuals within a cluster are identical and the sample size is the number of clusters. Larger cluster sizes are associated with smaller ICC values. In the study by Hillman et al. [5], the number of clusters was 23 and the ICC used was 0.02.

The study by Priestley et al. [12] was described as a step wedge CRCT. This involves a sequential roll-out of and intervention to clusters over a number of time periods [33]. The order in which clusters receive the intervention is random. The measurement of data from all clusters and at each step is a key feature that distinguishes the step wedge design from a classic cluster RCT [33, 34]. This trial did not measure at each step and is actually reported more like a cluster RCT [34]. We therefore calculated the design effect according to the procedure recommended by the Cochrane Collaboration [20].

Sensitivity analyses were pre-specified. The treatment effect was reported using a cumulative meta-analysis method fixed-effects model and a graphical augmentation to the funnel plot to assess the impact of a new study on the existing meta-analysis. An influence analysis was performed in which the pooled estimates are computed omitting one study at a time. Additionally a sensitivity analysis was conducted using an ICC of 0.01 and of 0.05 to establish whether this qualitatively changed results. The details of this analysis are included in Additional file 1. The analysis was performed using STATA statistical software (version 12.0; Statacorp, College Station, TX, USA) and Revman (version 5.1; Copenhagen: The Nordic Cochrane Centre, The Cochrane Collaboration).

## Results

A total of 29 publications were included in the analysis (Fig. 1). No unpublished studies were obtained.

### Study characteristics

The RRS studies had an effective sample size of 2,160,213 patients (1,107,492 in the intervention group and 1,108,380 in the control group) (Table 1). Nineteen studies (65.5 %) reported physicians as part of the RRS team for 24 hours per day and 7 days per week, two studies only had physician presence for office hours Monday to Friday, seven studies had no physician presence and one study did not report on the composition of the team. All of the studies have been published since 2000 and 13 studies have been published after 2008 (the end date for systematic review by Chan et al. [17]). Twenty-five studies were single centre. Twenty-one studies were conducted in academic hospitals, seven in community hospitals and one study used multiple sites that included both academic and community hospitals. The characteristics of the RRS intervention are described in Table 2. The number of RRS team activations per 1000 admissions was reported in 23 studies and varied substantially across studies. The mean and 95 % CI for the adult and paediatric activations per 1000 admissions were 16.3 (9.0–23.7) and 16.8 (6.0–27.6), respectively. About 33 % (95 % CI 23–43 %) of referrals were admitted to the ICU immediately after a RRS team consultation and 9.7 % (95 % CI 4.5–14.9 %) acquired a new designation of do not attempt resuscitation.

**Table 1:** Characteristics of included studies

ID	Author	Country	Year	Study design	Number and type of sites and population	Type of team
1	Al-Qahtani et al. [40]	Saudi Arabia	2013	Before/after without contemporaneous controls	1/academic/adult	1 ICU doctor
						1 ICU nurse
						1 ward nurse
						1 respiratory therapist
2	Baxter et al. [41]	Canada	2008	Before/after without contemporaneous controls	2/community/adult	1 ICU doctor
						1 ICU nurse
						1 respiratory therapist
3	Beitler et al. [42]	USA	2011	Before/after without contemporaneous controls	1/academic/adult	Medical doctor
						ICU nurse
						Respiratory therapist
						Patient transporter
4	Bellomo et al. [22]	Australia	2004	Before/after study without contemporaneous controls	1/academic/adult	1 ICU doctor
						1 Nurse
5	Bristow et al. [43]	Australia	2000	Controlled before/after	3/community/adult	1 ICU doctor
						1 general medicine doctor
						1 nurse
6	Buist et al. [44]	Australia	2002	Before/after without contemporaneous controls	1/academic/adult	2 doctors
						1 ICU nurse
7	Campello et al. [45]	Portugal	2009	Before/after without contemporaneous controls	1/community/adult	1 ICU doctor
						1 nurse
8	Chan et al. [13]	USA	2008	Before/after without contemporaneous controls	1/academic/adult	2 ICU nurses
						1 respiratory therapist
9	Dacey et al. [46]	USA	2007	Before/after without contemporaneous controls	1/community/adult	1 ICU or hospitalist doctor
						1 ICU nurse
						1 respiratory therapist
						1 physician assistant
10	De Vita et al. [47]	USA	2004	Before/after study without contemporaneous controls	1/academic/adult	1 ICU doctor
						1 anaesthetist
						2 physician
						2 ICU nurse
						1 ward nurse
11	Hayani et al. [48]	Canada	2011	Before/after without contemporaneous controls	1/academic/adult	1 ICU doctor
						1 ICU nurse
						1 respiratory therapist
12	Hillman et al. [5]	Australia	2004	Cluster RCT	23/mixed/adult	ICU or ED doctor
						ICU or ED nurse
13	Howell et al. [49]	USA	2012	Interrupted time series	1/academic/adult	Ward doctor
						2 nurses
						1 respiratory therapist
14	Jones et al. [50]	Australia	2005	Before/after without contemporaneous controls	1/academic/adult	ICU doctor
						ICU nurse
15	Kenward et al. [51]	UK	2004	Before/after study without contemporaneous controls	1/community/adult	NR
16	Konrad et al. [14]	Sweden	2010	Before/after without contemporaneous controls	1/academic/adult	1 ICU doctor
						1 ward doctor
						1 ICU nurse
						1 ward nurse
17	Lim et al. [52]	South Korea	2011	Before/after without contemporaneous controls	1/academic/adult	1 ICU doctor
						1 ICU nurse
						1 respiratory therapist
18	Priestley et al. [12]	UK	2004	Step wedge cluster RCT	1/community/adult	2 nurses
19	Santamaria et al. [53]	Australia	2010	Before/after without contemporaneous controls	1/academic/adult	1 ICU doctor
						1 general medicine doctor
						1 ICU nurse
20	Shah et al. [54]	USA	2011	Before/after without contemporaneous controls	2/academic/adult	1 ICU nurse 1 respiratory therapist
21	Simmes et al. [55]	The Netherlands	2012	Before/after without contemporaneous controls	1/academic/adult	1 ICU physician
						1 ICU nurse
22	Brilli et al. [56]	USA	2007	Before/after without contemporaneous controls	1/academic/paediatric	1 ICU doctor
						1 nurse
						1 respiratory therapist
23	Hanson et al. [57]	USA	2010	Interrupted time series	1/academic/paediatric	1 PICU doctor
						1 nurse
						1 respiratory therapist
24	Anwar ul Haque et al. [58]	Pakistan	2010	Before/after without contemporaneous controls	1/academic/paediatric	1 ICU doctor
25	Hunt et al. [59]	USA	2008	Before/after without contemporaneous controls	1/academic/paediatric	3 PICU doctors
						1 PICU nurse
						1 PICU respiratory therapist
26	Kotsakis et al. [60]	Canada	2011	Prospective before/after without contemporaneous controls	4/academic/paediatric	1 doctor (PICU daytime ICU night-time)
						1 PICU nurse
						1 respiratory therapist
27	Sharek et al. [61]	USA	2007	Before/after without contemporaneous controls	1/academic/paediatric	1 PICU doctor
						1 PICU or cardiac nurse
						1 respiratory therapist
28	Tibbals and Kinney [23]	Australia	2009	Before/after without contemporaneous controls	1/academic/paediatric	1 ICU doctor
						1 ED doctor
						1 general medicine doctor
						1 ICU nurse
29	Zenker et al. [62]	USA	2007	Before/after without contemporaneous controls	1/academic/paediatric	1 doctor
						1 PICU nurse
						1 respiratory therapist

*ED* emergency department, *NR* not reported, *PICU* paediatric intensive care unit, *RCT* randomized controlled trial

**Table 2:** Characteristics of rapid response system implementation and interventions

ID	Author	Calls per 1000 admissions	DNAR (%)	Control period/implementation period/intervention period (months)	ICU disposition (%)	Types of interventions (%)	Study definition of cardiac arrest and mortality
1	Al-Qahtani et al. [40]	18.2	9.3	24/0/36	40.2	Intubation 4	Non-ICU cardiopulmonary arrests, hospital mortality, including patients with DNAR designation
						NIV 8	
						IV fluids 48	
						Diuretics 13	
						Vasoactive infusions 5	
2	Baxter et al. [41]	40.3	8	24/12/12	27	Intubation 5	All cases of arrest Hospital-wide deaths, patients with DNAR designation included
						NIV 6	
						IV fluids 32	
						Diuretics 10	
						Vasopressors 8	
3	Beitler et al. [42]	10.8	11.2	36/0/36	43.4	NR	Non-ICU cardiopulmonary arrests, hospital mortality, patients with DNAR designation included
4	Bellomo et al. [22]	4.7	10	4/14/4	18.2	Intubation 3	All cardiac arrests, hospital mortality, patients with DNAR designation included
						NIV 9	
						IV fluids 18	
						Diuretics 11	
						Vasopressors 5	
5	Bristow et al. [43]	NR	NR	NR/NR/6	NR	NR	All cardiac arrests, hospital mortality, patients with DNAR designation included
6	Buist et al. [44]	6.7	10.5	12/24/12	10.5	NR	All cardiac arrests, hospital mortality, patients with DNAR designation included
7	Campello et al. [45]	7.8	NR	12/0/48	NR	NR	All cardiac arrests, hospital mortality, patients with DNAR designation included
8	Chan et al. [13]	15.1	2.1	20/4/20	41.2	Intubation 7	Hospital-wide cardiopulmonary arrest and mortality with DNAR designation included
						NIV 11	
						IV fluids 16	
						Diuretics 7	
						Vasopressors 1	
9	Dacey et al. [46]	20.1	10	4/1/12	24	Intubation 11	All cardiac arrests, and hospital mortality with DNAR designation included
						NIV 10	
						IV fluids 32	
10	De Vita et al. [47]	25.8	NR	60/0/20		NR	Hospital-wide cardiopulmonary arrest mortality not reported
11	Hayani et al. [48]	NR	NR	60/0/36	NR	NR	Mortality at 100 days after transplant
12	Hillman et al. [5]	8.7	8	2/4/6	30	NR	Non-ICU cardiopulmonary arrest Cardiac arrest and non-ICU mortality with DNAR designation excluded
13	Howell et al. [49]	53	NR	22/6/31	20	NR	Hospital mortality with DNAR designation excluded
14	Jones et al. [50]	25.2	NR	12/14/50	NR	NR	All cardiac arrests, and hospital mortality with DNAR designation included
15	Kenward et al. [51]	53	25	12/0/12	20	Intubation 23	All cardiac arrests, and hospital mortality with DNAR designation included
						IV fluids 25	
16	Konrad et al. [14]	2.5	26	60/3/24	27	NR	All cardiac arrests, and hospital mortality DNAR designation included
17	Lim et al. [52]	NR	NR	6/6/6	NR	NR	All cardiac arrests, and hospital mortality including DNAR designation
18	Priestley et al. [12]	NR	NR	0/0/8	NR	NR	Cardiac arrest NR; hospital mortality including DNAR designation
19	Santamaria et al. [53]	8.7	NR	30/3/18	NR	NR	All cardiac arrest and hospital mortality including DNAR designation included
20	Shah et al. [54]	26.7	7	9/6/27	50	NR	All cardiac arrest and hospital mortality with DNAR designation included
21	Simmes et al. [55]	56	NR	12/3/25	53	NR	All cardiac arrest and hospital mortality with DNAR designation excluded
23	Hanson et al. [57]	NR	NR	24/10/12	57	NR	Non-ICU cardiac arrest and hospital mortality with DNAR designation included
24	Anwar ul Haque et al. [58]	21	NR	10/0/9	39	Intubation 18	All cardiac arrest and hospital mortality with DNAR designation included
25	Hunt et al. [59]	11.9	NR	12/0/12	NR	NR	Non-ICU cardiac arrest, hospital mortality NR
26	Kotsakis et al. [60]	44.2	NR	24/9/24	30	NR	All cardiac arrest and hospital mortality with DNAR designation included
27	Sharek et al. [61]	19.7	0.7	54/2/19	57	Intubation 0.7	Non-ICU cardiac arrest and hospital mortality with DNAR designation included
						IV fluids 16	
						Vasopressors 4	
28	Tibballs and Kinney [23]	5.1	NR	41/3/48	47	Intubation 8	All cardiac arrest and hospital mortality with DNAR designation included
						NIV 7	
						IV fluids 23	
						Vasopressors 4	
29	Zenker et al. [62]	12.8	NR	23/0/12	36	Intubation 7	All cardiac arrest and hospital mortality with DNAR designation included
						NIV 6	
						IV fluids	
						Vasopressors 2	

*IV* intravenous fluids, *DNAR* do not attempt resuscitation, *NIV* non-invasive ventilation, *NR* not reported

### Risk of bias within studies

Two investigators (RM and IR) assessed study quality independently. The risk of bias for each study is presented in Tables 3, 4 and 5. The Newcastle Ottawa Quality Assessment Scale was used to evaluate the before–after studies without contemporaneous controls [35]. The interrupted time series, controlled before–after and cluster randomized studies were evaluated using the criteria recommended by the Cochrane Effective Practice and Organisation of Care Group [26]. This included recruitment bias, baseline imbalance, loss of clusters, incorrect analysis or selective outcome reporting.

**Table 3:** Risk of bias table for cluster randomized control trials and controlled before–after trials

Study	Allocation sequence generation	Allocation concealment	Baseline comparability	Complete outcome data	Outcome variables assessed blindly	Protection from contamination	Selective outcome reporting	Free from other biases
Bristow et al. [42]	High risk	High risk	Low risk	Low risk	Low risk	Low risk	Low risk	Low risk
Priestley et al. [12]	High risk	High risk	High risk	High risk	Unclear	Unclear	High risk	Low risk
Hillman et al. [5]	Low risk	Low risk	Low risk	Low risk	Low risk	Low risk	Low risk	Low risk

**Table 4:** Risk of bias table using the Newcastle Ottawa Quality Assessment Scale for cohort studies

Study	Selection				Comparability	Outcome		
	Representativeness of exposed cohort	Selection of non-exposed cohort	Ascertainment of exposure	Outcome of interest was not present at the start of the study	Comparability of cohorts on the basis of design or analysis	Assessment of outcome	Was follow-up long enough for the events to occur?	Adequacy of follow-up cohorts
Al-Qahtani et al. [40]	*	*	*	*		*	*	*
Baxter et al. [41]	*	*	*	*		*	*	*
Beitler et al. [42]			*	*		*	*	*
Bellomo et al. [22]	*	*	*	*	*	*	*	*
Buist et al. [44]	*	*		*	*	*	*	
Campello et al. [45]	*		*	*		*	*	*
Dacey et al. [46]	*	*	*	*	*	*		
DeVita et al. [47]	*	*	*	*				*
Hayani et al. [48]			*	*		*	*	
Jones et al. [50]	*	*	*	*		*	*	
Kenward et al. [51]	*	*	*	*	*	*	*	
Konrad et al. [14]	*	*	*	*	*	*	*	*
Lim et al. [52]	*	*	*	*		*		*
Santamaria et al. [53]	*	*	*	*	*	*	*	*
Shah et al. [54]	*	*	*	*	*	*	*	*
Simmes et al. [55]			*	*		*		
Brilli et al. [56]	*	*	*	*	*	*	*	*
Anwar ul Haque et al. [58]		*		*		*	*	
Hunt et al. [59]	*	*	*	*		*	*	*
Kotsakis et al. [60]				*	*		*	*
Sharek et al. [61]	*	*	*	*	*	*	*	*
Tibballs and Kinney [23]	*	*		*		*	*	*
Zenker et al. [62]		*		*		*	*	*

**Table 5:** Risk of bias table for interrupted time series studies

Study	Was the intervention independent of other changes?	Was the shape of the intervention effect pre-specified?	Was the intervention unlikely to affect data collection?	Was knowledge of the allocated interventions adequately prevented during the study?	Were incomplete outcome data adequately addressed?	Was the study free from selective outcome reporting?	Was the study free from other risks of bias?
Howell et al. [49]	Low risk	Low risk	Low risk	Low risk	Low risk	Low risk	Low risk
Hanson et al. [57]	Low risk	Unclear risk	Low risk	Low risk	Low risk	Low risk	Low risk

In general terms, the number of stars denotes study quality. A study can earn one star for each component of ‘Selection’, ‘Outcome’ and ‘Comparability’. Representativeness is awarded a star if the cohort is truly or somewhat representative of the population of interest. A star is awarded for selection of the non-exposed cohort, if it is drawn from the same population as the exposed cohort. Exposure is satisfactorily ascertained if data were acquired from secure records. A maximum of two stars can be given for ‘Comparability’. Either the exposed or non-exposed were matched in design or confounders adjusted in analysis. A maximum of three stars can be given for ‘Outcome’. Assessment of outcome is awarded a star if the outcomes were assessed by independent or blind assessment, confirmation of records by reference to secure records or record linkage. The adequacy of the duration of follow-up should be awarded a star if it was long enough for the outcomes to occur. Completeness of follow-up was considered adequate if losses were not related to the exposure or the outcome and was sufficiently low to be unlikely to introduce bias.

### Syntheses of results Primary outcome

#### Hospital mortality

**Fig. 2:**
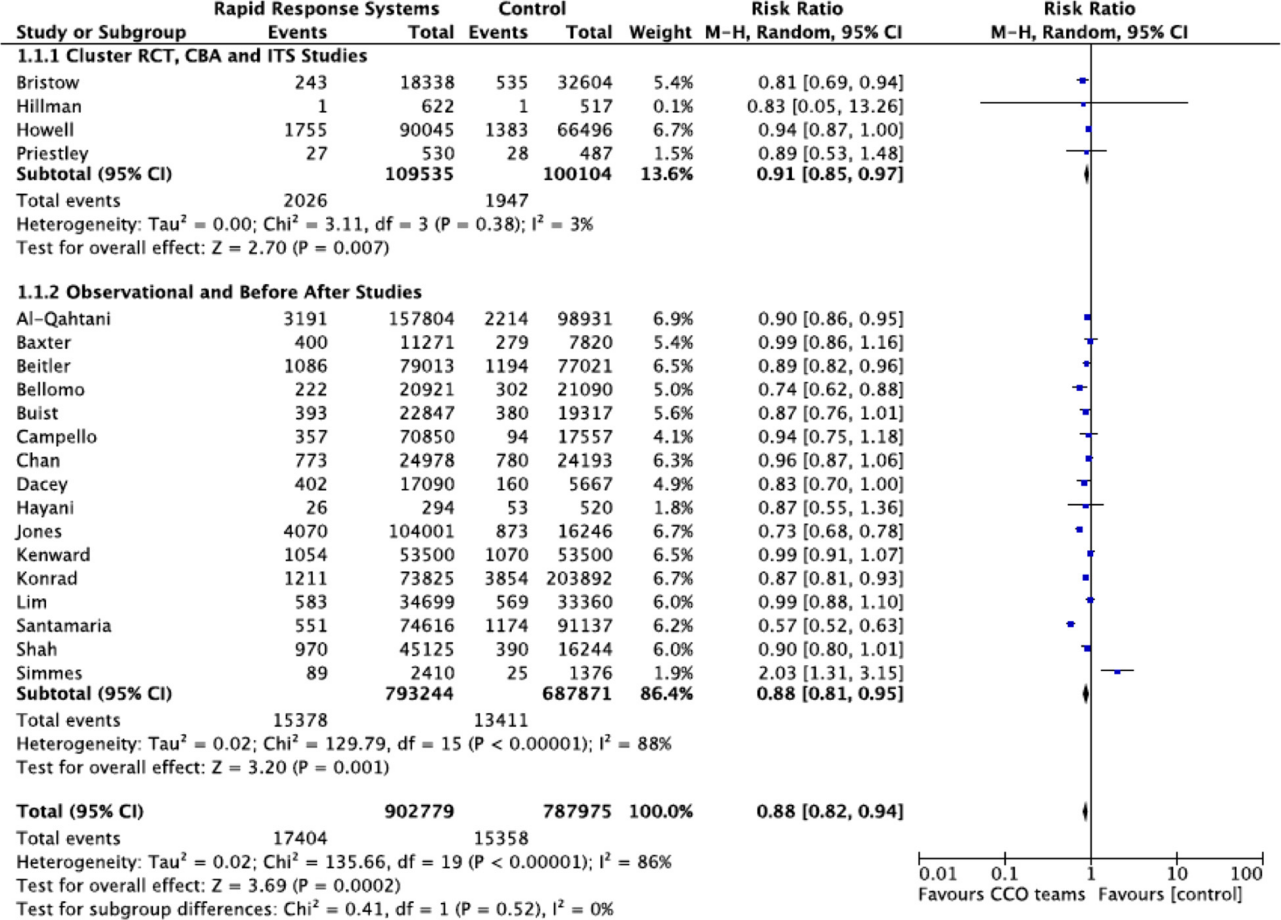
Forest plot of the effect of rapid response system teams on hospital mortality in adult in-patients. Weights are calculated from random-effects analysis. *CBA* controlled before–after, *CCO* critical care outreach, *CI* confidence interval, *ITS* interrupted time series, *RCT* randomized controlled trial

**Fig. 3:**
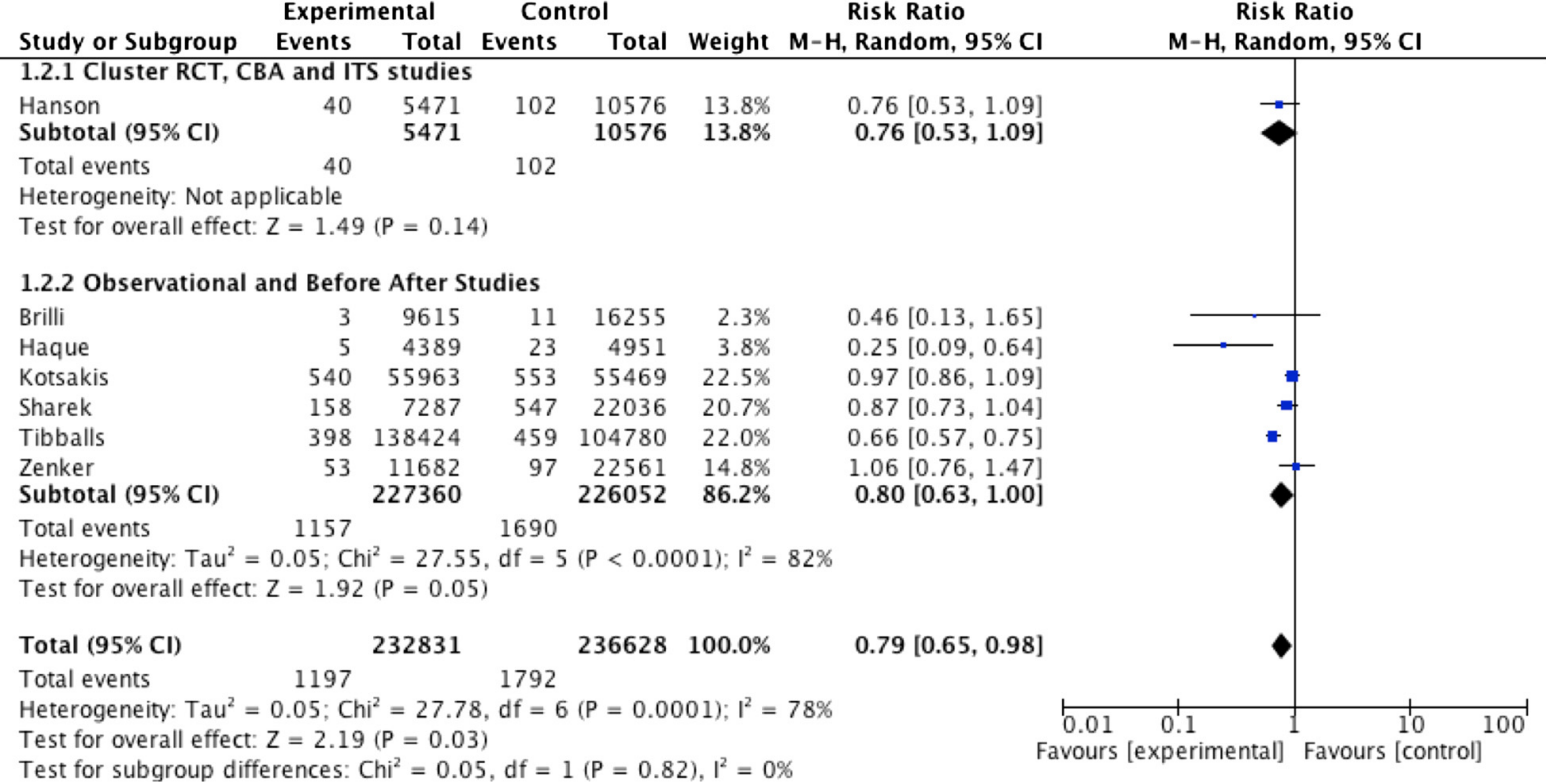
Forest plot of the effect of rapid response system teams on hospital mortality in paediatric in-patients. Weights are calculated from random-effects analysis. *CBA* controlled before–after, *CI* confidence interval, *ITS* interrupted time series, *RCT* randomized controlled trial

The implementation of RRS in the adult population has been associated with an overall reduction in hospital mortality (RR 0.87, 95 % CI 0.81–0.95, *p* <0.001) (Fig. 2). There was evidence of considerable heterogeneity (*I*^2^ = 86 %, *p* <0.001). The treatment effect in the cluster randomized trials, controlled before–after and interrupted time series studies was RR 0.91 (95 % CI 0.85–0.97) with less heterogeneity (*I*^2^ = 3 %). In the paediatric population, RRS also showed a reduction in mortality (RR 0.82, 95 % CI 0.76–0.89) with significant heterogeneity (*I*^2^ = 78 %) (Fig. 3). There was only one study in the cluster randomized control study, controlled before–after and interrupted time series subgroup so no subgroup analysis based on study design could be performed.

### Secondary outcomes

#### Cardiopulmonary arrests

The implementation of RRS in the adult population has been associated with an overall reduction in cardiopulmonary arrests (RR 0.65, 95 % CI 0.61–0.70, *p* <0.001) with substantial heterogeneity (*I*^2^ = 70 %, *p* <0.001) (Figure S1 in Additional file 1). The treatment effect in the cluster randomized trials, controlled before–after and interrupted time series studies subgroup was RR 0.74 (95 % CI 0.56–0.98) with less heterogeneity (*I*^2^ = 0 %). In the paediatric population, RRS also showed a reduction in cardiopulmonary arrests (RR = 0.64, 95 % CI 0.55–0.74) with minimal heterogeneity (*I*^2^ = 7 %) (Figure S2 in Additional file 1).

#### ICU admissions

Only 10 of the adult studies reported the effect of RRS teams on the number ICU admissions. The implementation of RRS in the adult population has not been associated with a significant effect on the number of ICU admissions (RR 0.90, 95 % CI 0.70–1.16, *p* = 0.43). None of the paediatric studies reported the effect of RRS teams on the number of ICU admissions.

### Assessment of publication bias

**Fig. 4:**
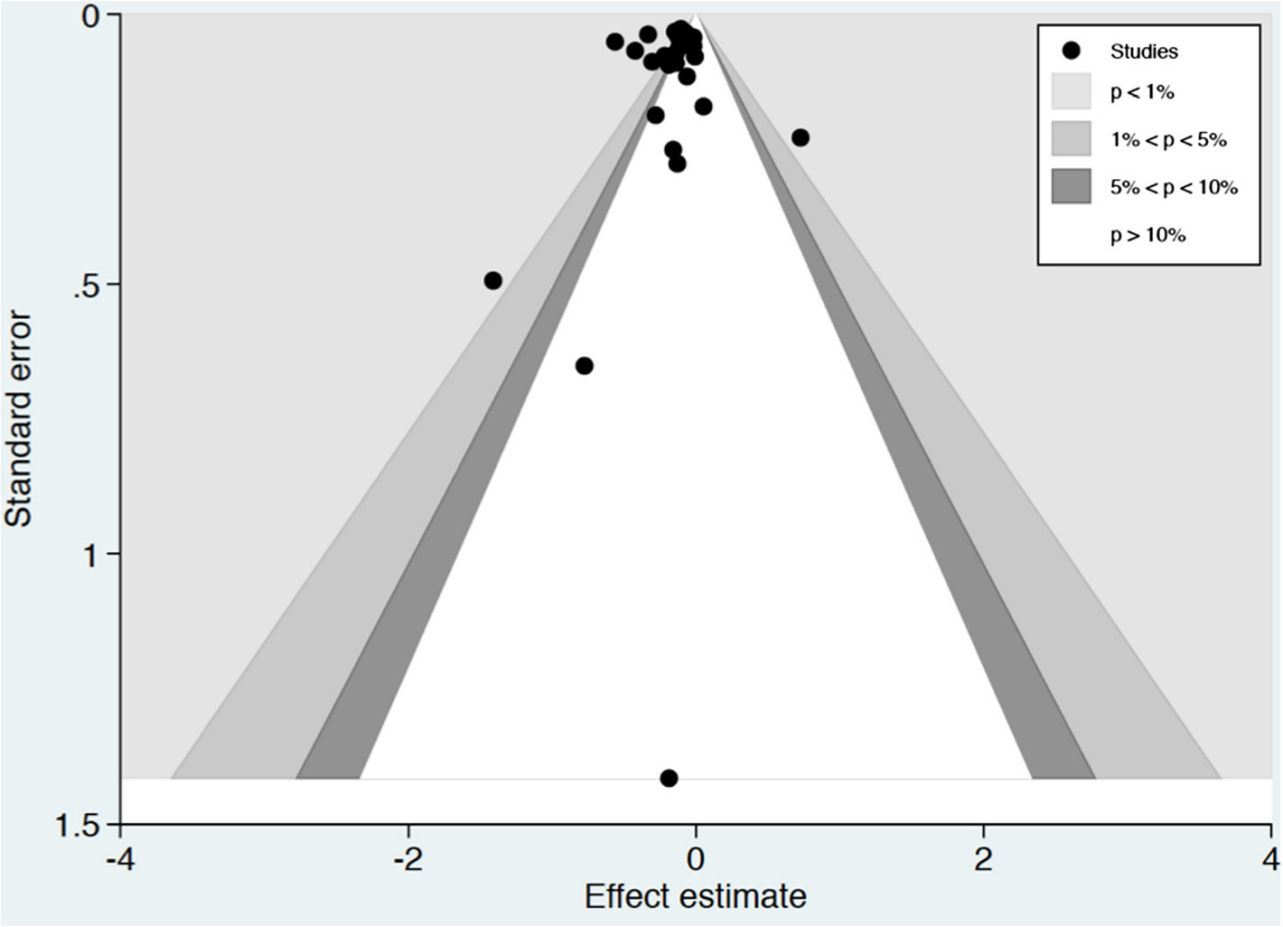
Contour-enhanced funnel plot. If studies appear to be missing in areas of low statistical significance, then it is possible that the asymmetry is due to publication bias. Conversely, if the area in which studies are perceived to be missing are of high statistical significance, then publication bias is a less likely cause of the funnel asymmetry

Publication bias refers to the phenomenon in which studies with less favourable results are less likely to be published than those with favourable results. Funnel plots appear asymmetric because of systematic suppression of studies. There are many factors other than publication bias that may explain funnel plot asymmetry, such as differential study quality or small study effects. A contour-enhanced funnel plot aims to disentangle these causes of funnel plot asymmetry. Generally, the level of statistical significance may drive publication bias, with studies that do not reach the perceived milestones of significance (e.g. *p* <0.05) less likely to be published. A contour-enhanced funnel plot overlays the contours of statistical significance on a funnel plot. This provides a novel method to assess whether the studies that exist are areas of statistical significance or whether there are areas where studies are missing that correspond to areas of low statistical significance. If studies are missing in areas of low statistical significance, then there may be publication bias. If studies are perceived to be missing, then publication bias is less likely. The contour-enhanced funnel plot was asymmetric but the perceived missing studies were in areas of high statistical significance, making publication bias a less likely cause of funnel asymmetry (Fig. 4).

The Harbord modification of the Egger test was used to assess funnel plot asymmetry. The estimated intercept was −0.207 with a standard error of 0.897 and a *p* value of 0.819. This result suggests little evidence for small study effects.

### Assessment of heterogeneity

A meta-regression was performed to explore the heterogeneity between studies. The covariates used were the number of activations per 1000 admissions, the duration of implementation in months and the presence of a physician in the RRS team. The model was adjusted for multiplicity using the Monte-Carlo permutation test for meta-regression. There was no significant relationship between any of the covariates (activation dose (*p* = 0.112), implementation time (*p* = 0.999) or physician presence (*p* = 0.992)) and hospital mortality. The model accounted for 18 % of the observed heterogeneity. The residual 82 % heterogeneity was probably due to persisting between study effects.

### Sensitivity analysis

Assumptions about the effects of clustering were explored using an ICC of 0 and of 0.01 (Figures S3 and S4 in Additional file 1). Neither of these assumptions changed the treatment effect and is included in the supplementary data. A sensitivity analysis was performed by systematically omitting individual studies to establish the influence on outcome (Figure S5 in Additional file 1). This showed that there was no study whose removal would materially change the pooled estimate of hospital mortality. A cumulative meta-analysis was performed in which the cumulative evidence at the time of each study is calculated (Figure S6 in Additional file 1). This analysis showed that the effect estimate has been consistent over time. A further analysis examined the potential impact a new study would have on the existing meta-analysis, providing an indication of the robustness of the results to the addition of new evidence. The graph shows that all studies lie in the region with a beneficial treatment effect, which dominates the graph (Figure S7 in Additional file 1). This result suggests that the meta-analysis is relatively robust to the addition of a single new trial.

## Discussion

In a systematic review and meta-analysis of 29 studies we found that a RRS team was associated with a reduction in hospital mortality in both adult and paediatric hospital populations. Our study reveals a striking degree of variation in how RRS teams were constituted, delivered and evaluated. The term ‘rapid response system’ may refer to rapid response teams, medical emergency teams or critical care outreach teams. Critical care outreach teams have the functionality of rapid response teams together with a surveillance function as well as ICU discharge follow-up.

A number of findings merit further discussion. There was a high degree of between-study heterogeneity in the included studies. The analysis included studies with different methodologies and could account for this observation. The adult CRCTs, controlled before–after and interrupted time series studies showed minimal heterogeneity (*I*^2^ = 3 %), compared with observational and before–after studies without a contemporaneous control (*I*^2^ = 88 %). This may be due to a small number of studies in the former subgroup.

This study did not find any dose–response relationship between the duration of the implementation phase, the presence of a physician on the team or the number of activations per 1000 and hospital mortality. These covariates were chosen because of pre-existing favourable reports [4]. Previous reports have suggested that a longer duration of implementation may lead to higher levels of support and engagement with the RRS [4, 36].

The optimal composition of the RRS team is uncertain. Two previous single-centre reports did not show the benefits of intensivist-led teams compared with registrar or resident-led teams [37, 38]. The majority of RRS interventions did not require the presence of a physician (fluids, oxygen and diuretics). Decisions around end-of-life planning may require physician involvement but would not necessarily manifest as changes in hospital mortality, although they could affect the number of cardiopulmonary resuscitation codes on the wards. It is possible that the presence of a physician in the team may have a differential effect in university hospitals compared with community hospitals, but there are insufficient data to establish this.

An increase in RRS team activations per 1000 admissions had previously been associated with reduction in cardiac arrest [39]. We were unable to show a significant relationship between RRS team activation and hospital mortality. This may suggest that the mechanism by which RSS teams reduce mortality is not through reductions in cardiac arrest. Very sensitive calling criteria may overactivate the RSS team, causing system fatigue with no tangible benefit. A final caveat is that the interpretation of meta-regression should always be undertaken cautiously. Meta-regression has limitations: the small number of studies, correlation between covariates and unmeasured characteristics, differences in the relationships that occur at a patient level and that may not be detected at a study level, and the tendency of regression to the mean.

The effect on the RRS team on the ICU workflow is important. The study was not able to show any effect on overall numbers of ICU admissions.

This study has several limitations. The vast majority of studies were observational studies without a contemporaneous control. Whilst there are several guidelines for the reporting of these studies, valuable information was often missing. The subgroup analysis did not find any significant difference in treatment effect in the different study methodologies. The outcomes of studies were reported variably. Some studies reported all hospital mortality and others reported only non-DNAR designated hospital mortality We used all hospital mortality reported because this offers the most conservative estimate of treatment. The major strength of our study is that the treatment effect has been consistent over time, is not influenced by any single study, and is robust to assumptions about clustering and to a further study being conducted.

## Conclusion

This study found that RRS teams associate with a reduction in hospital mortality and cardiac arrest. These findings did not show any significant publication bias. A sensitivity analysis showed that the study findings were robust to addition of a new study. We were unable to show any benefit from the presence of a physician on the RRS team, the duration of implementation or the number of activations. Whilst RRS teams are very much part of the landscape in many health systems, further work is needed to understand the specific factors that are likely to mitigate their effectiveness in given operational contexts.

### Key messages

- RRS teams are effective in reducing hospital mortality in both adult and paediatric in-patients.
- RRS teams also reduce hospital cardiac arrest.
- The vast majority of rapid response interventions do not require a physician and the presence of a physician was not associated with improved outcomes.

## Additional file

Additional file 1: Figure S1. Showing a forest plot of the effect of rapid response system teams on adult cardiac arrest, **Figure S2.** showing a forest plot of the effect of rapid response system teams on paediatric cardiac arrest, **Figure S3.** showing a forest plot of the effect of rapid response system teams on hospital mortality in adult in-patients using an ICC of 0.01, **Figure S4.** showing a forest plot of the effect of rapid response system teams on hospital mortality in adult in-patients using an ICC of 0, **Figure S5.** showing the influence of removing one study at a time on the pooled effect, **Figure S6.** showing the cumulative influence of study on meta-analysis of hospital mortality, and **Figure S7.** showing contours for areas in which new studies would have to lie for the pooled result to achieve significance at 5 %.

## Abbreviations

CI: confidence interval
CINAHL: Cumulative Index to Nursing and Allied Health Literature
CRCT: cluster randomized control trial
ICC: intracluster correlation coefficient
NICE: National Institute of Clinical Excellence
NOS: Newcastle Ottawa Scale
RR: risk ratio
RRS: rapid response system
