# Supplementary material for: Rapid response systems: a systematic review and meta-analysis
Source: Crit Care. 2015 Jun 12;19(1):254. doi: 10.1186/s13054-015-0973-y (PMC4489005; doi:10.1186/s13054-015-0973-y)
Supplement: Additional file 1: Figure S1. — Showing a forest plot of the effect of rapid response system teams on adult cardiac arrest, Figure S2. showing a forest plot of the effect of rapid response system teams on paediatric cardiac arrest, Figure S3. showing a forest plot of the effect of rapid response system teams on hospital mortality in adult in-patients using an ICC of 0.01, Figure S4. showing a forest plot of the effect of rapid response system teams on hospital mortality in adult in-patients using an ICC of 0, Figure S5. showing the influence of removing one study at a time on the pooled effect, Figure S6. showing the cumulative influence of study on meta-analysis of hospital mortality, and Figure S7. showing contours for areas in which new studies would have to lie for the pooled result to achieve significance at 5 %. [file 13054_2015_973_MOESM1_ESM.docx]

**Rapid Response Systems: A systematic review and Meta-Analysis**

Additional file

Contents

Figure S1. Forest plot of the effect of Rapid Response System teams on adult cardiac arrest.

Figure S2. Forest plot of the effect of Rapid Response System teams on paediatric cardiac arrest

Figure S3. Forest plot of the effect of Rapid Response System teams on hospital mortality in adult in-patients using an intra-cluster correlation coefficient of 0.01 .

Figure S4 Forest plot of the effect of Rapid Response System teams on hospital mortality in adult in-patients using an intra-cluster correlation coefficient of 0.

Figure S5. The influence of removing one study at a time on the pooled effect

Figure S6. Cumulative influence of study on meta-analysis of hospital mortality.

Figure S7. Contours for areas in which new studies would have to lie for the pooled result to achieve significance at 5% are presented

Figure S1. Forest plot of the effect of Rapid Response System teams on adult cardiac arrest. Weights calculated from random effects model.


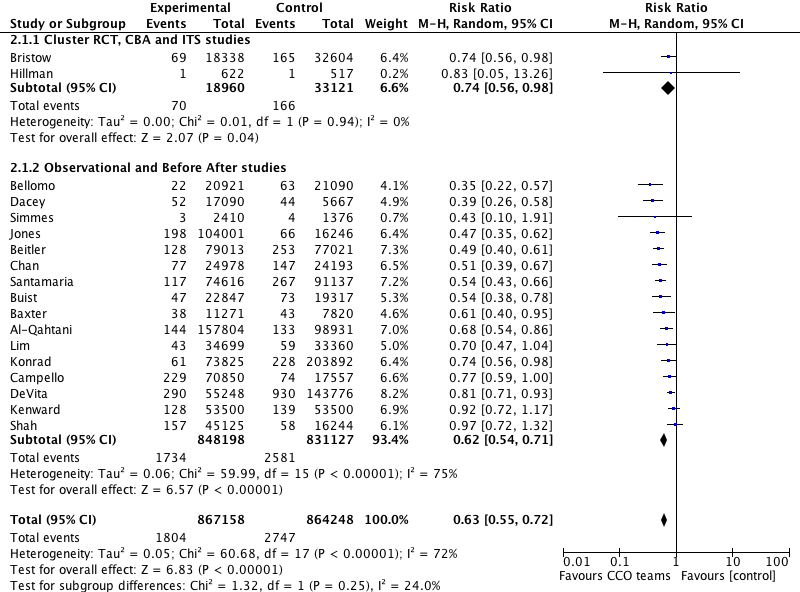


Figure S2. Forest plot of the effect of Rapid Response System teams on paediatric cardiac arrest. Weights calculated from random effects model.


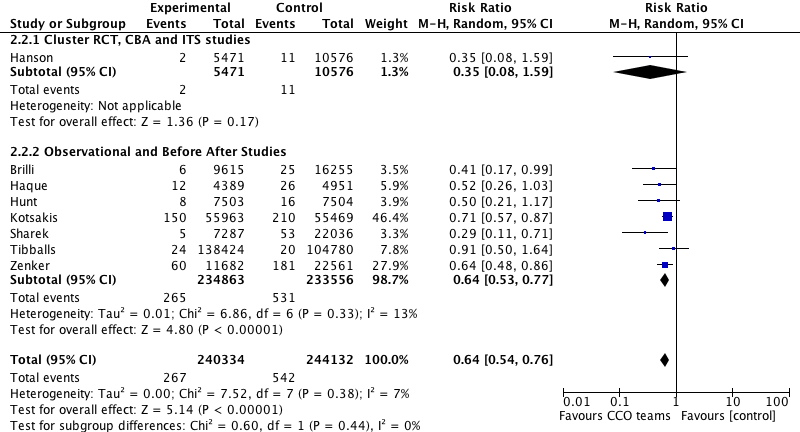


Figure S3. Forest plot of the effect of Rapid Response System teams on hospital mortality in adult in-patients using an intra-cluster correlation coefficient (ICC) of 0.01. Weights are calculated from random-effects analysis. (CI denotes confidence interval)


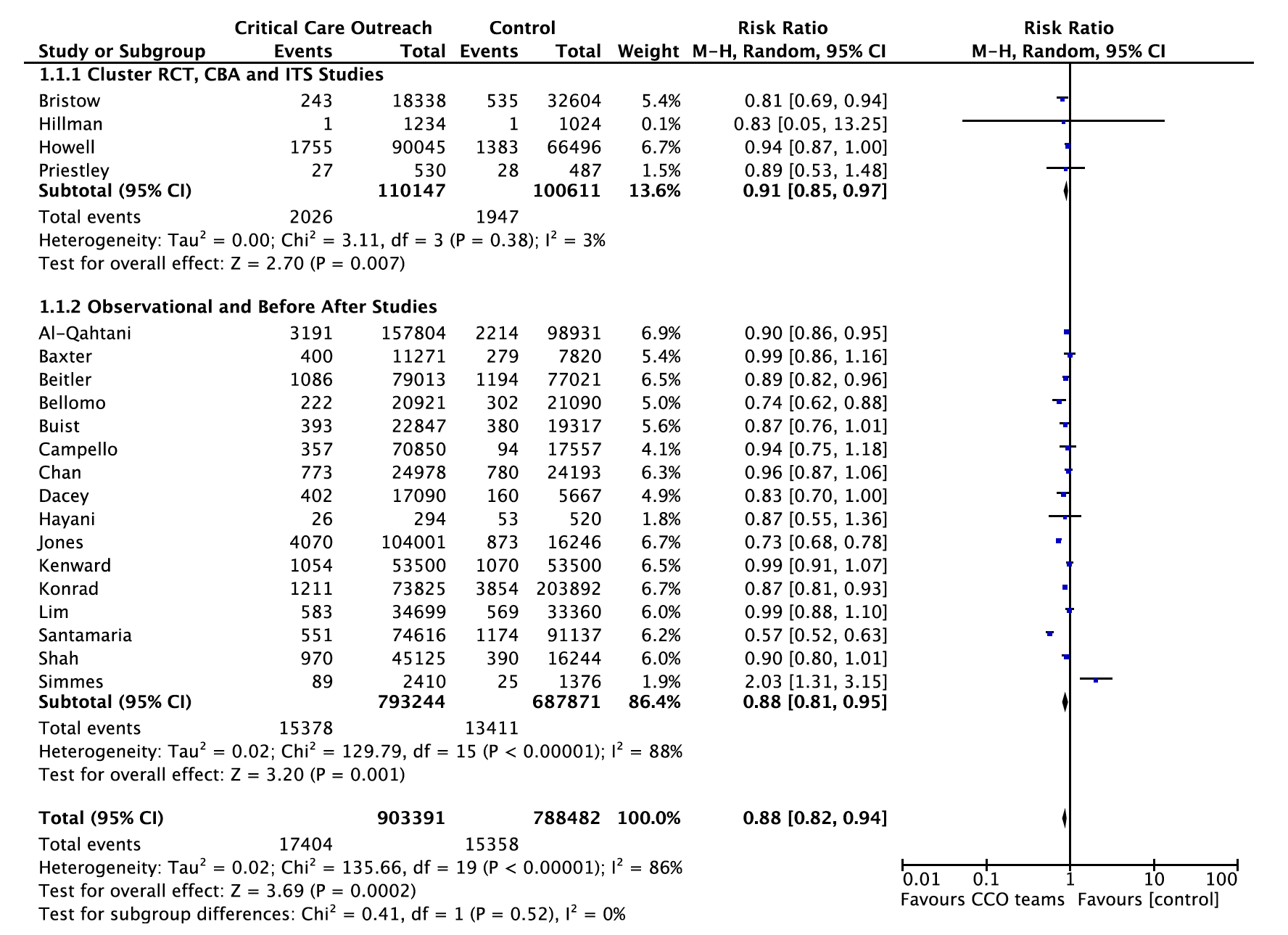


Figure S4. Forest plot of the effect of Rapid Response System teams on hospital mortality in adult in-patients using an intra-cluster correlation coefficient (ICC) of 0. Weights are calculated from random-effects analysis. (CI denotes confidence interval)


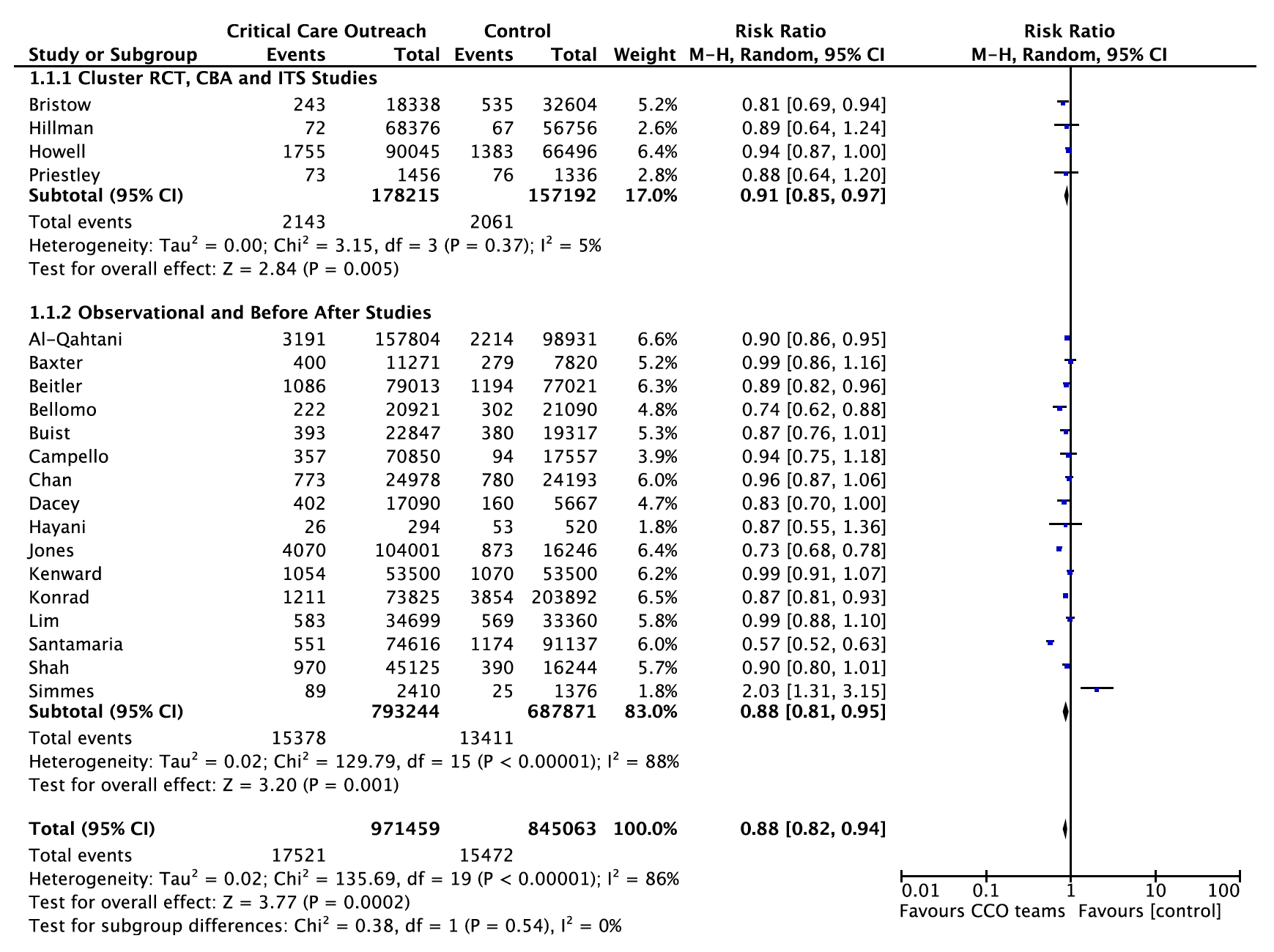


Figure S5. The influence of removing one study at a time on the pooled effect. The omission of any study does not have a significantly influence the overall effect.


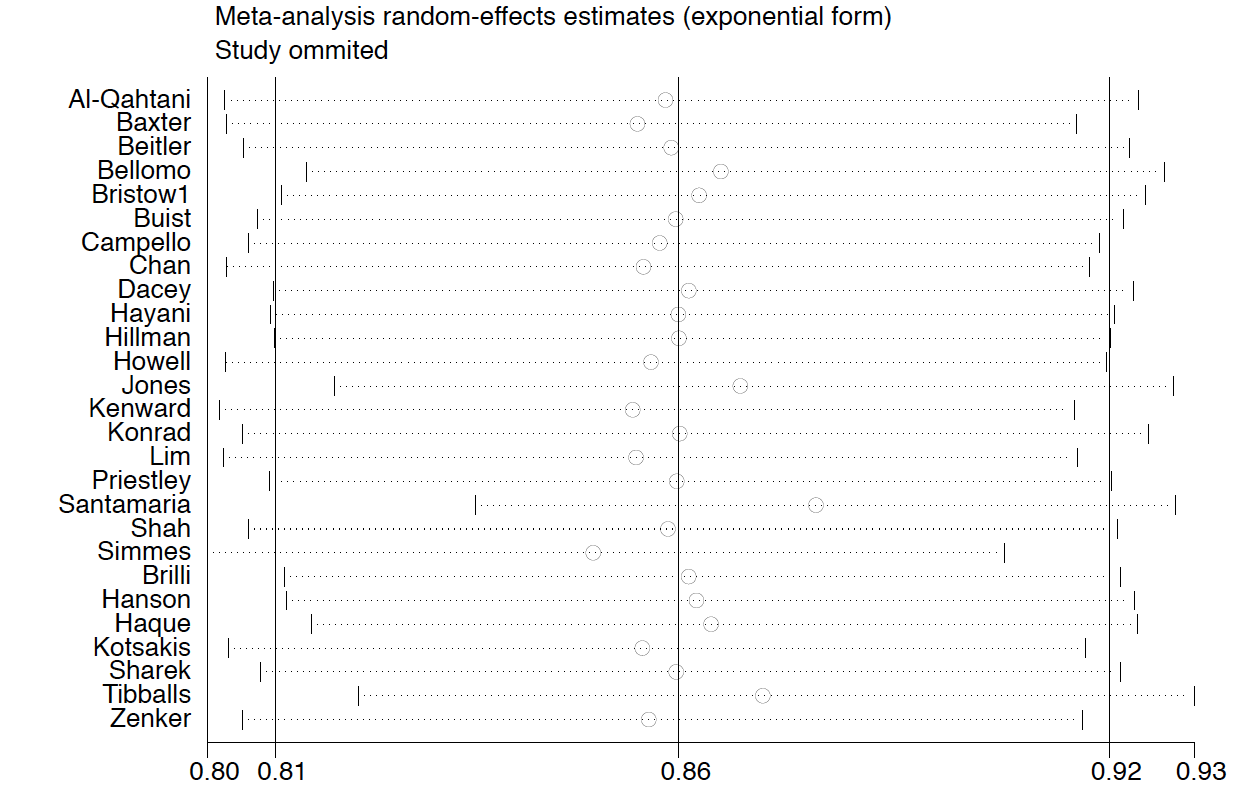


Figure S6. Cumulative influence of study on meta-analysis of hospital mortality. The vertical dashed line represents the pooled risk ratio of 0.88.


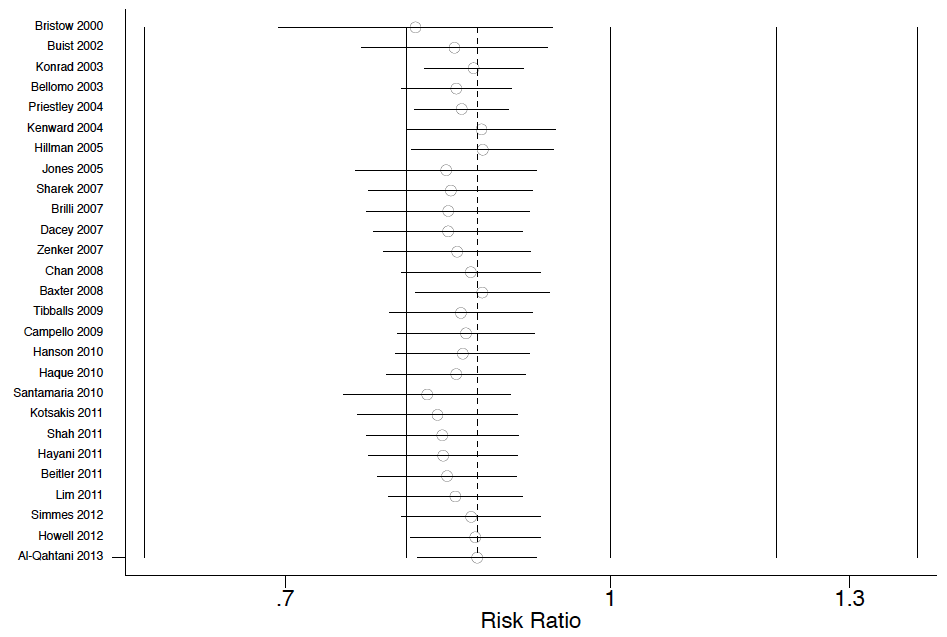


Figure S7. Contours for areas in which new studies would have to lie for the pooled result to achieve significance at 5% are presented. All studies lie in the region of statistical significance with beneficial treatment effect. The region of beneficial treatment effect dominates the graph and it makes the meta-analysis robust to the addition of an additional study.


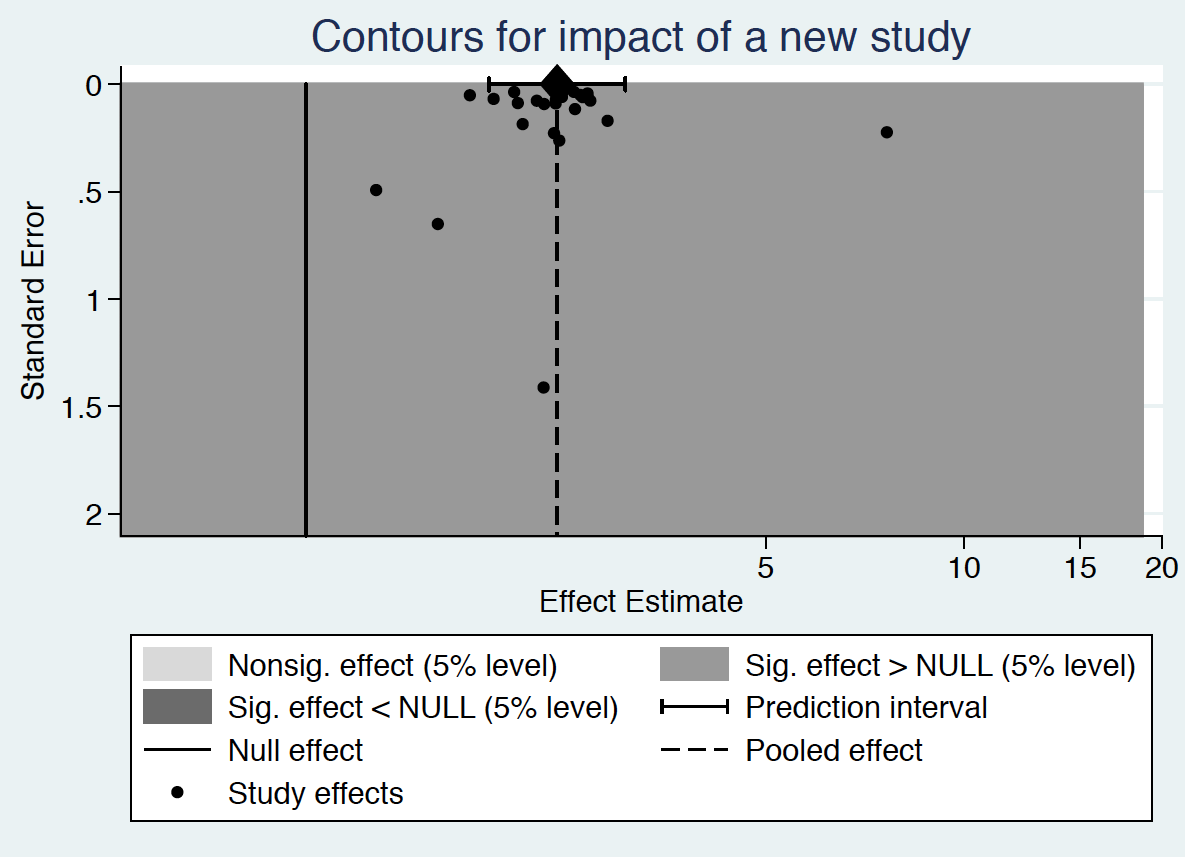


**SEARCH STRATEGY**:

rapid response team OR medical emergency team OR critical care outreach team

AND

effective* OR implement* OR success* OR fail* OR utiliz* OR adopt*

**COCHRANE:**

Records identified trough COCHRANE: 72

Records after duplicates removal: 72

Records excluded: 66 Not RSS study: 64

Not apply to key question (does RSS reduce mortality?): 2

Not original data:0

Not inpatient setting: 0

Not comparison group:0

Abstract only:0

Full-text assessed for eligibility: 6

Not pre-post study: 0

Included : 6

**CINHAL**

Records identified trough CINAHL: 361

Records after duplicates removal: 359

Records excluded: 340 Not RSS study: 54

Not apply to key question (does RSS reduce mortality?): 263

Not original data: 12

Not inpatient setting: 3

Not comparison group: 3

Abstract only: 6

Full-text assessed for eligibility: 18

Not pre-post study: 9

Included: 9

**EMBASE**

Records identified trough EMBASE: 1087

Records after duplicates removal: 1087

Records excluded: 1052 Not RSS study: 57

Not apply to key question (does RSS reduce mortality?): 943

Not original data:11

Not inpatient setting: 2

Not comparison group: 6

Abstract only: 33

Full-text assessed for eligibility: 35

Not pre-post study: 10

Included : 25

**PubMED**

Records identified trough PubMed: 1415

Records after duplicates removal: 1415

Records excluded: 138 Not a RRS study: 1233

Not apply to key question (does RSS reduce mortality?): 107

Not original data: 24

Not inpatient setting: 1

Not comparison group: 6

Abstract only: 0

Full-text assessed for eligibility: 44

Not pre-post study: 16

Included : 28

**TOTALLY INCLUDED FROM DIFFERENT DATABASES**: 68

**DUPLICATE REMOVED**: 39

**ELIGIBLE FOR METANALYSIS: 29**
